# Trajectories of hepatic and coagulation dysfunctions related to a rapidly fatal outcome among hospitalized patients with dengue fever in Tainan, 2015

**DOI:** 10.1371/journal.pntd.0007817

**Published:** 2019-12-05

**Authors:** Chun-Yin Yeh, Bing-Ze Lu, Wei-Jie Liang, Yu-Chen Shu, Kun-Ta Chuang, Po-Lin Chen, Wen-Chien Ko, Nai-Ying Ko

**Affiliations:** 1 Department of Computer Science and Information Engineering, National Cheng Kung University, Tainan, Taiwan; 2 Department of Nursing, College of Medicine, National Cheng Kung University, Tainan, Taiwan; 3 Department of Mathematics, National Cheng Kung University, Tainan, Taiwan; 4 Department of Artificial Intelligence, CTBC Business School, Tainan, Taiwan; 5 Department of Internal Medicine, College of Medicine, National Cheng Kung University, Tainan, Taiwan; 6 Department of Microbiology and Immunology, College of Medicine, National Cheng Kung University, Tainan, Taiwan; 7 Department of Public Health, College of Medicine, National Cheng Kung University, Tainan, Taiwan; Fundacao Oswaldo Cruz, BRAZIL

## Abstract

**Background:**

Hepatic dysfunction and coagulopathy are common in acute dengue illness. We analyzed the trajectories of the above parameters in the survivors and fatal patients in the outbreak in Tainan, 2015.

**Methods:**

A retrospective study was conducted using data from a tertiary hospital between January and December 2015. Multilevel modeling (MLM) was used to identify the changes in aminotransferase (AST), alanine aminotransferase (ALT), activated partial thromboplastin time (aPTT), and platelet counts from Day 0 to Day 7 of the onset of dengue infection. The machine-learning algorithm was used by purity measure assumption to calculate the accuracy of serum transaminases and coagulation variables to discriminate between the fatal and survival groups.

**Results:**

There were 4,069 dengue patients, of which 0.9% died in one week after illness onset (*i*.*e*., early mortality). Case fatality rate was the highest for those aged ≥70 years. Both AST and ALT values of the fatal group were significantly higher than those of the survivor group from Day 3 (AST median, 624 U/L vs. 60 U/L, *p* < 0.001; ALT median, 116 U/L vs. 29 U/L, *p* = 0.01) of illness onset and peaked on Day 6 (AST median, 9805 U/L vs. 90 U/L, *p* < 0.001; ALT median, 1504 U/L vs. 49 U/L, *p* < 0.001). AST ≥ 203 U/L, ALT ≥ 55 U/L, AST^2^/ALT criteria ≥337.35, or AST/platelet count ratio index (APRI) ≥ 19.18 on Day 3 of dengue infection had a high true positive rate, 90%, 78%, 100%, or 100%, respectively, of early mortality. The platelet counts of the fatal group declined significantly than those of the survivor group since Day 3 of illness onset (median, 19 x10^3^/μl vs. 91 x10^3^/μl, *p* < 0.01), and aPTT values of the fatal group significantly prolonged longer since Day 5 (median, 68.7 seconds vs. 40.1 seconds, *p* < 0.001).

**Conclusions:**

AST, ALT, and platelet counts should be monitored closely from Day 0 to Day 3 of dengue infection, and aPTT be followed up on Day 5 of infection to identify the individuals at risk for early mortality.

## Author summary

Dengue fever (DF) is currently one of the most severe public health problems. Clinical presentations of dengue are diverse and non-specific, often with unpredictable clinical progression and outcome. Hepatic dysfunction and abnormal coagulation factors are common in acute dengue illness, reflected by abnormal alanine aminotransferase (AST), aspartate aminotransferase (ALT), activated partial thromboplastin time (aPTT), and platelet counts. However, there is no information available about the monitoring frequency required, which could help identify those dengue patients who are likely to die, especially during epidemic outbreaks with limited healthcare resources. We examined all the laboratory-confirmed dengue patients who admitted to the major tertiary hospital in Tainan during the 2015 dengue outbreak, and the different trajectories of hepatic function and coagulation factors between survivors and rapidly fatal dengue patients were analyzed. Although there were no differences in AST, ALT, aPTT, and platelet counts between the survivor and fatal groups on the day DF symptoms first appeared, the differences increased from the early stages of infection and became more prominent during the early stages of the illness. The necessity of monitoring the AST, ALT, aPTT, and platelet count frequently during the febrile phase is emphasized by this study.

## Introduction

Dengue incidence has risen 30-fold in the past fifty years globally, with the South-East Asia and Western Pacific Regions being the most affected regions with an approximately 1% fatality rate [1]. In 2015, Taiwan experienced one of the most severe dengue outbreaks in history with 43,832 cases, including 228 deaths [2]. Among these dengue cases, 22,777 (52%) were reported in Tainan and resulted in 189 deaths, and led to struggles triaging and managing patients with dengue fever (DF) in the primary care setting [3]. Without timely triage and management, dengue patients run increased risks of developing severe dengue resulting in death, which usually coincides with the critical phase (days 3–7) of the illness [1]. However, the manifestations of dengue vary that results in the difficulties to predict the progress of dengue patients clinically [1]. It is important to develop an effective method for identifying the objective risk factors for patients who become potentially fatal early in the course of dengue infection, which can optimize patient follow-up and referral strategies in an area with limited resources during an epidemic outbreak [4].

Hepatic dysfunction and abnormal coagulation are common during the acute stage of dengue infection. About 61%–96% of dengue patients have elevated liver enzyme levels of aspartate aminotransferase (AST) and alanine transaminase (ALT), which peak at around day 7 of the onset of the illness [5, 6], while 7%–42.3% have prolonged activated partial thromboplastin time (aPTT) [5, 7] and 85.8%–98% have thrombocytopenia [8, 9]. Elevated liver transaminases [6, 9–12], prolonged aPTT [9, 12–14] and lower platelet count [4, 8, 9, 12, 13, 15] are more prominent in patients with severe dengue or poor outcomes. Several studies have tried to identify the correlation of hepatic dysfunction and abnormal coagulation with poor outcomes, which includes increased severity of vascular permeability and bleeding complications [16, 17], admission to an intensive care unit [18], a prolonged hospital stay [7, 13] and mortality [7, 14, 19]. However, studies have presented inconsistent findings. The liver aminotransferase levels lack discriminatory function to classify the severity of dengue [10] and are not independent predictors of mortality [20]. Moreover, the mean platelet volume shows no correlation with severe dengue [8]. This inconsistency may result from the risk factors only being assessed at a single time-point such as at the time of admission and the day prior to poor outcomes, or only examining the peak values during treatment, which made these markers less relevant to the progress of illness [21]. Therefore, the most accurate prediction of prognosis would be achieved by using longitudinal sequential data collected frequently during the course of the illness [4].

Limited studies have included the involvement of liver function and coagulation factors in their data collection. The investigators in Vietnam [17] determined transaminase levels at intervals during the course of dengue and showed that both AST and ALT values began to rise from an early stage before increasing significantly during the critical phase, and were correlated with disease severity. A later study in Sri Lanka [6] showed AST values were much higher on days 5 and 6 of the illness in patients with severe dengue infection. Furthermore, one prospective observational study in Vietnam [4] showed that changes in the platelet count over time were related to developing severe dengue, and emphasized the necessity of monitoring laboratory data daily during the early stage of dengue, which corresponds to the suggestion from the World Health Organization (WHO) [22]. However, the clinical implementation of daily testing over a long period time would be difficult due to the high utilization of healthcare resources during epidemic outbreaks. In addition, there is no information available about the monitoring frequency required, which could help identify those dengue patients who are likely to die.

This study analyzed the trajectories of hepatic function and coagulation factors between survivors and fatal dengue patients who were treated at a tertiary hospital during the 2015 outbreak in Tainan. Our research findings provide supportive evidence that the frequent assessment of hepatic and coagulation markers can help to identify the patients with higher risk to be rapidly fatal.

## Methods

### Ethics statement

The study protocol was approved by the Institutional Review Board (IRB), National Cheng Kung University Hospital (NCKUH) (A-ER-104-386). The IRB waived the need for informed consent, and all data analyzed were anonymized.

### Study design and study population

A retrospective study was conducted using 20,213 laboratory test results from 4,069 patients who were diagnosed with DF at NCKUH, a major tertiary hospital in Tainan city, between January and December 2015. Only those dengue patients who had at least one of the following WHO laboratory diagnosis criteria confirmed by Taiwan Centers of Disease Control (Taiwan CDC) were enrolled in this study: (1) viral isolation and serotype identification; (2) a positive reverse transcriptase-polymerase chain reaction (RT-PCR) (TIB Molbiol, Lightmix kit; Roche Applied Science, Berlin, Germany); (3) a positive nonstructural antigen 1 (NS1) reaction; (4) a positive enzyme-linked immunosorbent assay for specific immunoglobulin M (IgM) or IgG antibodies for dengue virus in their blood serum during the acute phases; (5) IgM seroconversion in paired sera or IgG seroconversion in paired sera or fourfold IgG titer increase in paired sera during the acute phases and convalescent stage [22, 23]. Serum from patients with suspected dengue infection was determined using the one-step immunochromatographic Dengue Duo Dengue NS1 Ag + Ab Combo assay (SD BIOLINE, Yongin, Korea).

### Data collection and definitions

All DF patients were divided into two groups according to whether they died within one week after the onset of illness or not (fatal group and survivor group). The management of different classification of dengue was evaluated at hospital admission in clinical practice according to the WHO criteria [22]. In this study, the trajectories of hepatic and coagulation markers of the fatal and survivor groups were the main explanatory variables. Hepatic markers were determined using a biochemical assay analyzer (Cobas 8000 c 702 module; Roche Diagnostics GmbH, Mannheim, Germany). Coagulation markers were tested using a Coulter LH 750 Hematology Analyzer (Beckman Coulter, Inc., California, USA). The values of AST, ALT, aPTT, and platelet counts in dengue-affected patients from Day 0 to Day 7, a total of 8 days, of the onset of illness were collected for analysis. Day 0 was defined as the date DF symptoms presented in these patients, which was determined by the reporting data from Taiwan CDC. Co-morbidities were defined as the diseases being recorded with ICD-9 codes among dengue patients admitted to NCKUH between January and December in 2015 (ICD-9 code: 070.3x, 070.5x for viral hepatitis B or C; 280.x–289.x for hematological disorders; 303.x for alcohol dependence; 401.x–405.x for hypertensive disease, 570.x–573.x for acute hepatitis or liver failure).

### Data analysis

The differences between fatal and survivor groups were examined using *t*-test, the Mann–Whitney *U* test, and the chi-squared test. Multilevel modeling (MLM) was used to identify the values of change in hepatic and coagulation markers from Day 0 to Day 7 for fatal and survivor groups. *P*-value and 95% confidence interval were considered significant if ≤0.05. MLM accounts for multiple observations of the same dengue patient in the current study, and a multilevel model is needed to determine if mean values of laboratory results vary notably across patients. The intraclass correlation coefficient (ICC) was used to explain a variation in laboratory results occurs across patients. All statistical analysis was conducted by using R Version 3.4.1 (Geneva, Switzerland).

The accuracy of the laboratory criteria for identifying early mortality was calculated using machine-learning. The criteria (c) were defined as the significant values among AST, ALT, AST^2^/ALT, aPTT, and AST/platelet count ratio index (APRI), the proved predictor of severe dengue [9, 16], in each day with the highest true positive rate (TPR) and the lowest false positive rate (FPR) to predict the mortality among dengue patients. The machine-learning algorithm was used through purity measure assumption, which was set as ||(*FPR*(*c*), *TPR*(*c*)) − (0,1)||_2_, to train the criteria to minimize the measure. Finally, the receiver operating characteristic curves (ROC) were constructed to evaluate the accuracy of the criteria to discriminate between fatal and survivor groups.

## Results

### Study population

All the patients (n = 4,069) who were diagnosed with DF at NCKUH were included in this study. Thirty-seven (0.9%) of these patients died within 8 days of the onset of the illness. Mortality was highest for those aged between 90–99 years (3 patients [14.29%]) (S1 Fig). Of these fatal cases, 51.4% died within 5 days of the onset of illness. The maximum number of deaths in one day, which was nine, occurred on Day 5 of the onset of the illness (S2 Fig). Fatal patients were older, more likely to be hospitalized, and with higher AST values at D0 than survivors (Table 1).

**Table 1:** Characteristics and initial laboratory data at the onset day of dengue fever (D0) among 4,069 patients.

Variables	Fatal patients, n = 37	Surviving patients, n = 4,032	*p* values
Age, years	74.9±12.0	47.8±21.5	<0.001
Male gender	14 (37.8%)	2039 (50.6%)	0.12
Days between illness onset and diagnosis	1 (0–5)	1 (0–67)	0.05
Days between illness onset and death	4 (0–7)	---	
Days between diagnosis and death	2 (-1-7)	---	
Ever hospitalization after the illness onset	24 (64.9%)	534 (13.2%)	<0.001
Diagnosis of viral hepatitis B or C ^a^	0 (0%)	13 (0.3%)	1.00
Diseases of hematological disorders ^a^	0 (0%)	215 (5.4%)	0.26
Diagnosis of alcohol dependence ^a^	0 (0%)	1 (0%)	1.00
Diagnosis of hypertensive disease ^a^	8 (27.6%)	420 (10.6%)	0.05
Diagnosis of acute hepatitis or liver failure ^a^	0 (0%)	62 (1.6%)	1.00
Aspartate transaminase (U/L)^b^	185 (47–1,481)	35 (17–1,019)	<0.001
Alanine transaminase (U/L)^c^	33.5 (10–357)	16 (10–491)	0.10
Activated partial thromboplastin time (seconds)^d^	39.7 (30.9–60.8)	38 (27.5–93.2)	0.65
Platelet (x10^3^/μl)^e^	113 (19–228)	166 (5–567)	0.38

Note: Data are expressed as case number (%), mean ± standard deviation, median (range)

^a^ Twenty-nine records from 29 patients in the fatal group and 3,971 records from 3,971 patients in the survivor group

^b^ Six test results from six patients in the fatal group and 529 test results from 526 patients in the survivor group were provided at D0.

^c^ Sixteen test results from sixteen patients in the fatal group and 1,105 test results from 1,097 patients in the survivor group were provided at D0.

^d^ Seven test results from seven patients in the fatal group and 306 test results from 305 patients in the survivor group were provided at D0.

^e^ Three test results from three patients in the fatal group and 546 test results from 542 patients in the survivor group were provided at D0.

### Trajectories of hepatic function within 8 days of DF onset

**Fig 1:**
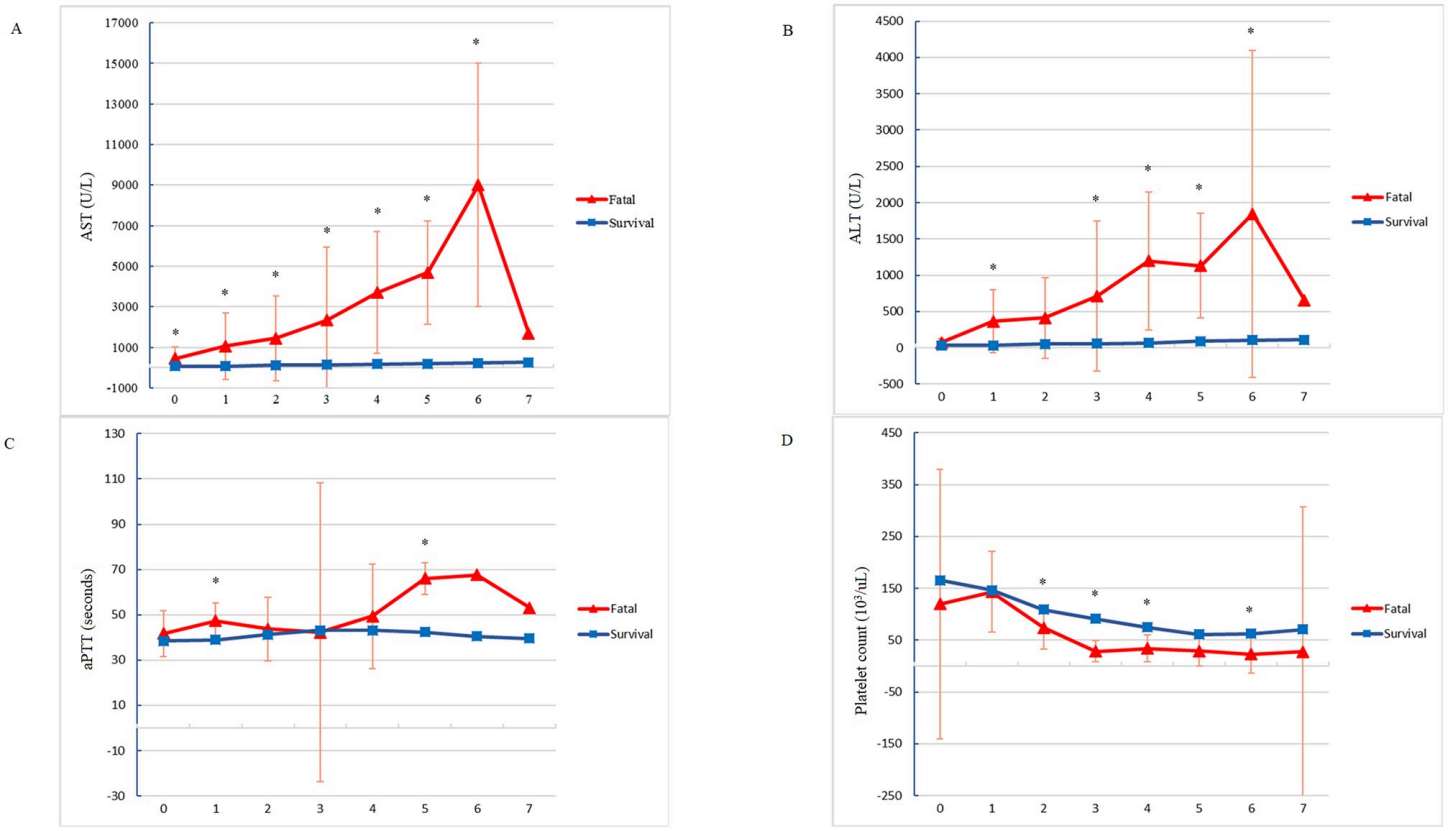
Trend variations in serum aspartate transaminase (AST) (Panel A), alanine transaminase (ALT) (Panel B), activated partial thromboplastin time (aPTT) (Panel C), and platelet count (Panel D) of the fatal and surviving patients with dengue fever within 8 days of illness onset.

Panel A and B in Fig 1 show serial AST and ALT values within 8 days of DF onset. MLM indicating that variance at the patient level accounted for 32% and 35% of the total variance of the AST and ALT values within 8 days of the onset of illness. In the fatal group, the AST and ALT values increased within 8 days of DF onset when compared with those of survivors after controlling of other comorbidities, including viral hepatitis B or C, hematological disorders, hypertensive disease, acute hepatitis, and liver failure (β = 2,812.72 for AST and β = 560.39 for ALT, both *p* values <0.001) (S1 Table). The Mann–Whitney *U* test showed a difference in laboratory manifestations between fatal and survivor groups on each day (S2 Table). The levels of hepatic enzymes in the fatal group were significantly higher than those of the survivor group, especially on Day 6 of the illness (fatal group: median, 9,805 U/L [range, 2,393–14,774 U/L] for AST; median, 1,504 U/L [range, 377–4,940 U/L] for ALT, both *p* values < 0.001).

### Trajectories of coagulation factors within 8 days of DF onset

Panel C and D of Fig 1 show the trajectories of aPTT and platelet counts within 8 days of DF onset. MLM indicating that variance at the patient level accounted for 14% and 59% of the total variance of the aPTT and platelet counts within 8 days of the onset of illness. In the fatal group, the aPTT became significantly prolonged than the survivor group after adjustment other cofounding factors (β = 12.18, *p* < 0.001) (S1 Table). In addition, the platelet counts in the fatal group decreased significantly, but not in the survivor group (β = −25.2, *p* = 0.01) (S1 Table). The Mann–Whitney *U* test showed that the largest difference in aPTT values between fatal and survivor groups occurred on Day 5 of the onset of the illness (median, 68.7 seconds [range, 52.2–82.1 seconds] in the fatal group vs. median, 40.1 seconds [range, 24–78.9 seconds] in the survivor group, *p* < 0.001) (S2 Table). The difference in platelet counts between fatal and survivor groups was most significant on Day 3 of the onset of the illness (median, 19 x10^3^/μl [range, 9–70 x10^3^/μl] in the fatal group vs. median, 91 x10^3^/μl [range, 4–325 x10^3^/μl] in the survivor group, *p* = 0.003) (S2 Table).

### Accuracy of hepatic and coagulation function for identifying mortality within 8 days of DF onset

**Fig 2:**
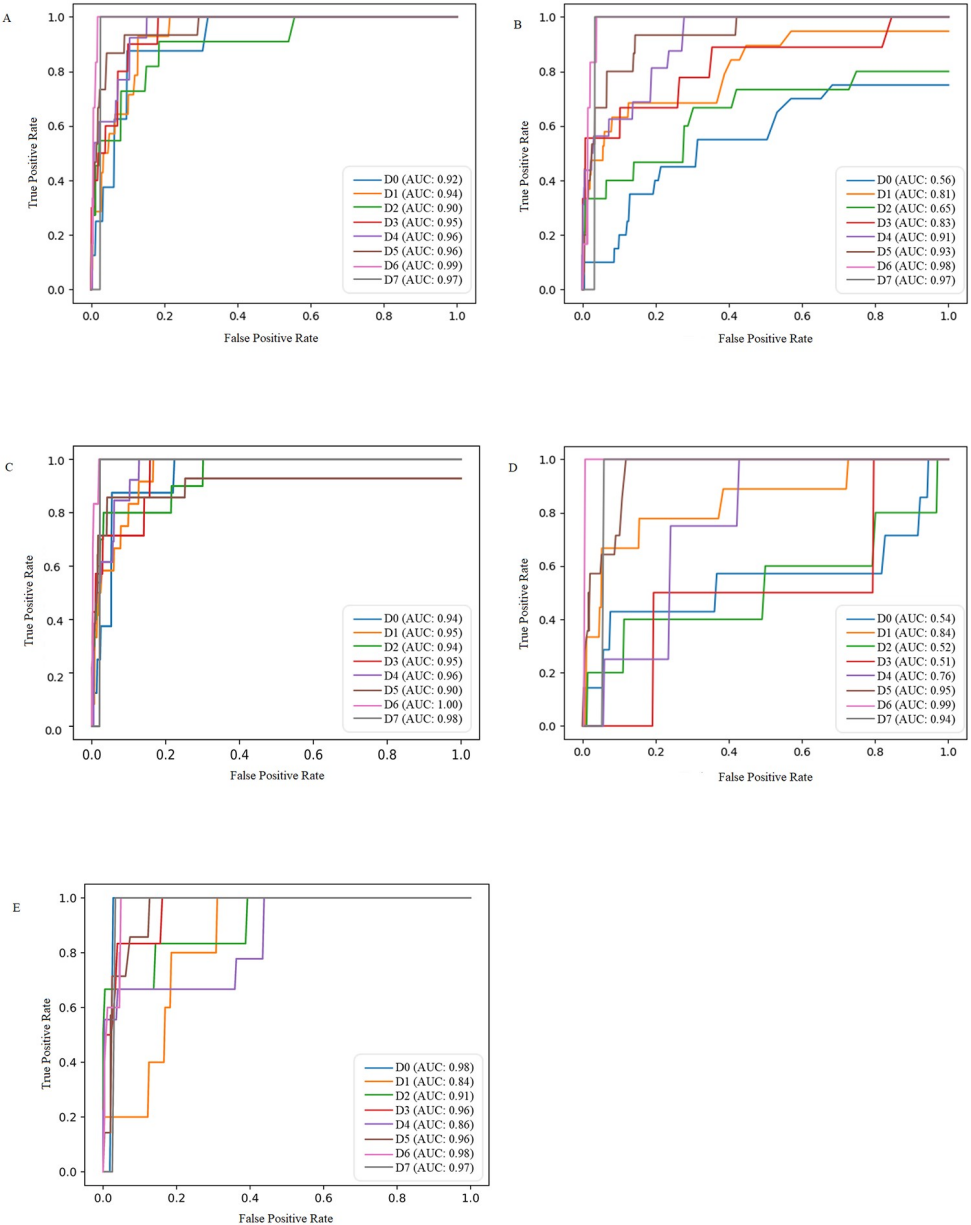
ROC of serum aspartate transaminase (AST) (Panel A), alanine transaminase (ALT) (Panel B), AST^2^/ALT (Panel C), activated partial thromboplastin time (aPTT) (Panel D), and AST/platelet count ratio index (APRI) (Panel E) in patients with dengue fever within 8 days of illness onset.

Fig 2 shows the ROC curves in AST (Panel A), ALT (Panel B), AST^2^/ALT (Panel C), aPTT (Panel D), and APRI (Panel E) in each day among dengue patients. Using AST criteria ≥ 203 U/L on Day 3 of dengue infection to identify mortality within 8 days of DF onset resulted in TPR of 90% and an area under the ROC curve (AUC) of 95%, while ALT criteria ≥ 55 U/L on Day 3 of dengue infection resulted in TPR of 78% and AUC of 83%. When considering both AST and ALT in AST^2^/ALT criteria ≥337.35 on Day 3 of dengue infection, the TPR was 100% and AUC was 95% (S3 Table). Using the criteria of aPTT ≥ 52 seconds on Day 5 after DF onset, the TPR of 100% and AUC of 95%. When considering both hepatic and coagulation factors in APRI criteria ≥ 19.18 on Day 3 of dengue infection, the probability of detecting mortality was 100% (S3 Table).

## Discussion

To the best of our knowledge, this is the first study to investigate longitudinal sequential data and identify the criteria for hepatic and coagulation function through machine learning within the early stage of acute dengue infection between fatal and survivor groups. Most of the dengue patients died from profound and prolonged shock resulting from plasma leakage, which was complicated by bleeding or fluid overload [22]. These conditions were reported in association with abnormal levels of AST and ALT [17], low platelet counts [4, 15, 19], and prolonged aPTT [7, 14]. In current study, we found that both AST and ALT values of the fatal group were significantly higher than those in the survivor group from Day 3 of illness and peaked on Day 6, which agrees with previous studies conducted in Sri Lanka [6] and Brazil [24]. Moreover, this study provides the evidence that AST ≥ 203 U/L, ALT ≥ 55 U/L, AST^2^/ALT criteria ≥ 337.35, and APRI ≥ 19.18 on Day 3 of illness resulted in high TPR for identification of early mortality. A previous study that tried to identify the changes of liver enzyme throughout the illness suggested not monitoring liver transaminase levels during the earlier stages of dengue infection due to the possible underestimation of liver injury [6]. However, the potential mechanisms of liver involvement during dengue infection, which include direct viral or host immune response of hepatocytes (Kupffer cells), is well established by the critical phase [5, 12, 17]. In addition, if abnormal liver enzymes persist during the critical phase of dengue infection, there is a higher possibility of progress to a life-threatening situation such as severe shock [5, 22]. Thus, monitoring the dynamic changes in liver function prior to the critical phase of dengue infection may provide early indicators of the acute exacerbation of DF. Our results highlighted the necessity of checking AST and ALT values consecutively from Day 0 to Day 3 of dengue infection to triage potentially fatal patients during the early stages of the illness.

This study also found that aPTT values of the fatal group were significantly prolonged over those of the survivors during Day 5 of the onset of illness, and the platelet counts of the fatal group decreased more significantly than those of the survivors from Day 3 of the onset of DF. The criteria of aPTT ≥ 52 seconds resulted in 100% TPR for identification of early mortality. Studies analyzing the initial admission laboratory data conducted in Taiwan [14] and Pakistan [7] demonstrated that aPTT prolongation was a prognostic factor for mortality. This might be explained partially by the dengue virus nonstructural protein NS1, which can bind to prothrombin and inhibit prothrombin activation [25]. Although the exact mechanisms of prolonged aPTT in dengue mortality remain unclear, our study findings provide evidence that prolonged aPTT contributes to catastrophic dengue infection. In contrast to the limited studies on aPTT, there have been many investigations of the relationship between the daily platelet count and the poor prognosis of dengue [4, 15]. In agreement with our findings, a study in Vietnam [4] also found that the platelet nadir occurred on day 6 of the illness, and suggested it is necessary to monitor the platelet count daily during the febrile phase of dengue [15]. However, considering the limited resources available during an epidemic outbreak, our results provide evidence that aPTT should be determined on Day 5 of dengue infection as aPTT prolongation was the most significant marker among the patients of the fatal group on Day 5 of infection. Based on our findings, platelet counts should be monitored during the first 4 days of infection because the differences in platelet counts between the fatal and survivor groups were most significant on Day 3 of the onset of illness.

This study has some limitations, which relate mainly to the analysis of secondary data. We were not able to determine other potentially relevant risk factors such as severe complications, primary or secondary infection status, the details of treatment regimens, and causes of death during dengue infection exactly, which means that our conclusion can only highlight the relationship between these vital markers and rapidly fatal dengue patients. Moreover, the current study was restricted to patients admitted to a single hospital. The laboratory data available was limited as there was no standardized protocol for monitoring hepatic and coagulation markers, which varied from one physician to another. However, it is still worth noting that the current results provide a reference for primary health care providers to follow up on vital markers related to rapidly fatal DF during the early stages of the illness. Another limitation of this study is that the exact mechanisms and interactions underlying the changes in AST, ALT, aPTT, and platelet counts remains obscure. Further studies will be necessary to develop reliable dynamic prediction models via the collection of comprehensive longitudinal information in the clinical setting.

In conclusion, the necessity of frequently monitoring AST, ALT, aPTT, and platelet counts during the febrile phase of DF is emphasized in this study. These findings reinforce the view that the incorporation of dynamic sequential data could help primary health care providers identify potentially fatal dengue patients during the early stages of infection. Further studies using abundant clinical information are warranted to clarify the mechanisms and relationships between abnormal hepatic function and coagulation factors and rapidly fatal DF.

## Supporting information

S1 Checklist STROBE Checklist. (DOCX)

S1 Fig Case fatality rate of patients with dengue fever among different age groups in the study hospital, 2015. (TIF)

S2 Fig Numbers and percentages of fatal cases of dengue fever in each day after illness onset in the study hospital, 2015. (TIF)

S1 Table Factors associated with trajectories of aspartate aminotransferase (AST), alanine transaminase (ALT), activated partial thromboplastin time (aPTT), platelet values of DF patients within 8 days after illness onset. (DOCX)

S2 Table The difference of aspartate aminotransferase (AST), alanine transaminase (ALT), activated partial thromboplastin time (aPTT), and platelet values between fatal and survivor group on each day. (DOCX)

S3 Table Characteristics of aspartate aminotransferase (AST), alanine transaminase (ALT), AST^2^/ALT, activated partial thromboplastin time (aPTT), and aspartate aminotransferase/platelet count ratio index (APRI) at selected criteria in each day. (DOCX)
