# Supplementary material for: Trajectories of hepatic and coagulation dysfunctions related to a rapidly fatal outcome among hospitalized patients with dengue fever in Tainan, 2015
Source: PLoS Negl Trop Dis. 2019 Dec 5;13(12):e0007817. doi: 10.1371/journal.pntd.0007817 (PMC6894745; doi:10.1371/journal.pntd.0007817)
Supplement: S1 Checklist — (DOCX) [file pntd.0007817.s001.docx]

STROBE Statement—checklist of items that should be included in reports of observational studies

|  | Item No. | Recommendation | Page  No. | Relevant text from manuscript |
| --- | --- | --- | --- | --- |
| **Title and abstract** | 1 | (*a*) Indicate the study’s design with a commonly used term in the title or the abstract | Abstract, “Methods” paragraph 2 (p.3) | A retrospective study was conducted using data from a tertiary hospital between January and December 2015. |
|  |  | (*b*) Provide in the abstract an informative and balanced summary of what was done and what was found | Abstract (p.3, 4) |  |
| Introduction | | | |  |
| Background/rationale | 2 | Explain the scientific background and rationale for the investigation being reported | Introduction, paragraph 3 (p.7,8) | However, the clinical implementation of daily testing over a long period time would be difficult due to the high utilization of healthcare resources during epidemic outbreaks. In addition, there is no information available about the monitoring frequency required, which could help identify those dengue patients who are likely to die. |
| Objectives | 3 | State specific objectives, including any prespecified hypotheses | Introduction, paragraph 4 (p.8) | This study analyzed the trajectories of hepatic function and coagulation factors between survivors and fatal dengue patients who were treated at a tertiary hospital during the 2015 outbreak in Tainan. Our research findings provide supportive evidence that the frequent assessment of hepatic and coagulation markers can help to identify the patients with higher risk to be rapidly fatal. |
| Methods | | | |  |
| Study design | 4 | Present key elements of study design early in the paper | Methods, paragraph 2 (P.9) | A retrospective study was conducted using 20,213 laboratory test results from 4,069 patients who were diagnosed with DF at NCKUH, a major tertiary hospital in Tainan city, between January and December 2015. |
| Setting | 5 | Describe the setting, locations, and relevant dates, including periods of recruitment, exposure, follow-up, and data collection | Methods, paragraph 2 (P.9) | A retrospective study was conducted using 20,213 laboratory test results from 4,069 patients who were diagnosed with DF at NCKUH, a major tertiary hospital in Tainan city, between January and December 2015. |
| Participants | 6 | (*a*) *Cohort study*—Give the eligibility criteria, and the sources and methods of selection of participants. Describe methods of follow-up  *Case-control study*—Give the eligibility criteria, and the sources and methods of case ascertainment and control selection. Give the rationale for the choice of cases and controls  *Cross-sectional study*—Give the eligibility criteria, and the sources and methods of selection of participants | Methods, paragraph 2, 3 (P.9,10) | A retrospective study was conducted using 20,213 laboratory test results from 4,069 patients who were diagnosed with DF at NCKUH, a major tertiary hospital in Tainan city, between January and December 2015. Only those dengue patients who had at least one of the following WHO laboratory diagnosis criteria confirmed by Taiwan Centers of Disease Control (Taiwan CDC) were enrolled in this study. All DF patients were divided into two groups according to whether they died within one week after the onset of illness or not (fatal group and survivor group). |
|  |  | (*b*) *Cohort study*—For matched studies, give matching criteria and number of exposed and unexposed  *Case-control study*—For matched studies, give matching criteria and the number of controls per case |  |  |
| Variables | 7 | Clearly define all outcomes, exposures, predictors, potential confounders, and effect modifiers. Give diagnostic criteria, if applicable | Methods, paragraph 3 (P.10) | The values of AST, ALT, aPTT, and platelet counts in dengue-affected patients from Day 0 to Day 7, a total of 8 days, of the onset of illness were collected for analysis. |
| Data sources/ measurement | 8* | For each variable of interest, give sources of data and details of methods of assessment (measurement). Describe comparability of assessment methods if there is more than one group | Methods, paragraph 3 (P.10) | The trajectories of hepatic and coagulation markers of the fatal and survivor groups were the main explanatory variables. |
| Bias | 9 | Describe any efforts to address potential sources of bias |  |  |
| Study size | 10 | Explain how the study size was arrived at | Methods, paragraph 2 (P.9) | Only those dengue patients who had at least one of the following WHO laboratory diagnosis criteria confirmed by Taiwan Centers of Disease Control (Taiwan CDC) were enrolled in this study. |

Continued on next page

| Quantitative variables | 11 | Explain how quantitative variables were handled in the analyses. If applicable, describe which groupings were chosen and why | Methods, paragraph 3 (P.10) |  |
| --- | --- | --- | --- | --- |
| Statistical methods | 12 | (*a*) Describe all statistical methods, including those used to control for confounding | Methods, paragraph 4 (P. 10) | The differences between fatal and survivor groups were examined using t-test, the Mann–Whitney U test, and the chi-squared test. Multilevel modeling (MLM) was used to identify the values of change in hepatic and coagulation markers from Day 0 to Day 7 for fatal and survivor groups. |
|  |  | (*b*) Describe any methods used to examine subgroups and interactions | Methods, paragraph 4 (P. 10) | Multilevel modeling (MLM) was used to identify the values of change in hepatic and coagulation markers from Day 0 to Day 7 for fatal and survivor groups. |
|  |  | (*c*) Explain how missing data were addressed |  |  |
|  |  | (*d*) *Cohort study*—If applicable, explain how loss to follow-up was addressed  *Case-control study*—If applicable, explain how matching of cases and controls was addressed  *Cross-sectional study*—If applicable, describe analytical methods taking account of sampling strategy |  |  |
|  |  | (*e*) Describe any sensitivity analyses | Methods, paragraph 4 (P. 10) | Finally, the receiver operating characteristic curves (ROC) were constructed to evaluate the accuracy of the criteria to discriminate between fatal and survivor groups. |
| Results | | | | |
| Participants | 13* | (a) Report numbers of individuals at each stage of study—eg numbers potentially eligible, examined for eligibility, confirmed eligible, included in the study, completing follow-up, and analysed | Results, paragraph 1 (P. 13) | All the patients (n=4,069) who were diagnosed with DF at NCKUH were included in this study. |
|  |  | (b) Give reasons for non-participation at each stage |  |  |
|  |  | (c) Consider use of a flow diagram |  |  |
| Descriptive data | 14* | (a) Give characteristics of study participants (eg demographic, clinical, social) and information on exposures and potential confounders | Results, paragraph 1 (P. 13) | Patients who died were older, were more likely to have been hospitalized following the onset of DF and had significantly higher AST values the day DF symptoms presented than survivors (Table 1). |
|  |  | (b) Indicate number of participants with missing data for each variable of interest | Results, paragraph 1 (P. 14,15) | Table 1 |
|  |  | (c) *Cohort study*—Summarise follow-up time (eg, average and total amount) | Results, paragraph 1 (P. 13) | Of these fatal cases, 51.4% died within 5 days of the onset of illness. The maximum number of deaths in one day, which was nine, occurred on Day 5 of the onset of the illness (S2 Fig). |
| Outcome data | 15* | *Cohort study*—Report numbers of outcome events or summary measures over time | Results, paragraph 1 (P. 13) | Thirty-seven (0.9%) of these patients died within 8 days of the onset of the illness. Of these fatal cases, 51.4% died within 5 days of the onset of illness. The maximum number of deaths in one day, which was nine, occurred on Day 5 of the onset of the illness (S2 Fig). |
|  |  | *Case-control study—*Report numbers in each exposure category, or summary measures of exposure |  |  |
|  |  | *Cross-sectional study—*Report numbers of outcome events or summary measures |  |  |
| Main results | 16 | (*a*) Give unadjusted estimates and, if applicable, confounder-adjusted estimates and their precision (eg, 95% confidence interval). Make clear which confounders were adjusted for and why they were included | Results, paragraph 2, 3 (P. 16) |  |
|  |  | (*b*) Report category boundaries when continuous variables were categorized |  |  |
|  |  | (*c*) If relevant, consider translating estimates of relative risk into absolute risk for a meaningful time period |  |  |

Continued on next page

| Other analyses | 17 | Report other analyses done—eg analyses of subgroups and interactions, and sensitivity analyses | Results, paragraph 4 (P. 17) | Accuracy of hepatic and coagulation function for identifying mortality within 8 days of DF onset |
| --- | --- | --- | --- | --- |
| Discussion | | | | |
| Key results | 18 | Summarise key results with reference to study objectives | Discussion, paragraph 1,2 (P. 19, 20) | In current study, we found that both AST and ALT values of the fatal group were significantly higher than those in the survivor group from Day 3 of illness, and peaked on Day 6.  This study also found that aPTT values of the fatal group were significantly prolonged over those of the survivors during Day 5 of the onset of illness, and the platelet counts of the fatal group decreased more significantly than those of the survivors from Day 3 of the onset of DF. The criteria of aPTT ≥ 52 seconds resulted in 100% TPR for identification of early mortality. |
| Limitations | 19 | Discuss limitations of the study, taking into account sources of potential bias or imprecision. Discuss both direction and magnitude of any potential bias | Discussion, paragraph 3 (P. 20,21) | This study has some limitations, which relate mainly to the analysis of secondary data. |
| Interpretation | 20 | Give a cautious overall interpretation of results considering objectives, limitations, multiplicity of analyses, results from similar studies, and other relevant evidence | Discussion, paragraph 1,2,3 (P. 19,20,21) |  |
| Generalisability | 21 | Discuss the generalisability (external validity) of the study results | Discussion, paragraph 3 (P. 21) | Moreover, the current study was restricted to patients admitted to a single hospital. The laboratory data available was limited as there was no standardized protocol for monitoring hepatic and coagulation markers, which varied from one physician to another. |
| Other information | |  | | |
| Funding | 22 | Give the source of funding and the role of the funders for the present study and, if applicable, for the original study on which the present article is based | Financial Disclosure section of the submission form | This work was supported by the Ministry of Science and Technology, Taiwan (https://www.most.gov.tw/en/public, grant number: MOST 107-2634-F-006-001). |

*Give information separately for cases and controls in case-control studies and, if applicable, for exposed and unexposed groups in cohort and cross-sectional studies.

**Note:** An Explanation and Elaboration article discusses each checklist item and gives methodological background and published examples of transparent reporting. The STROBE checklist is best used in conjunction with this article (freely available on the Web sites of PLoS Medicine at http://www.plosmedicine.org/, Annals of Internal Medicine at http://www.annals.org/, and Epidemiology at http://www.epidem.com/). Information on the STROBE Initiative is available at www.strobe-statement.org.
