# Supplementary figures and images for: Trajectories of hepatic and coagulation dysfunctions related to a rapidly fatal outcome among hospitalized patients with dengue fever in Tainan, 2015
Source: PLoS Negl Trop Dis. 2019 Dec 5;13(12):e0007817. doi: 10.1371/journal.pntd.0007817 (PMC6894745; doi:10.1371/journal.pntd.0007817)

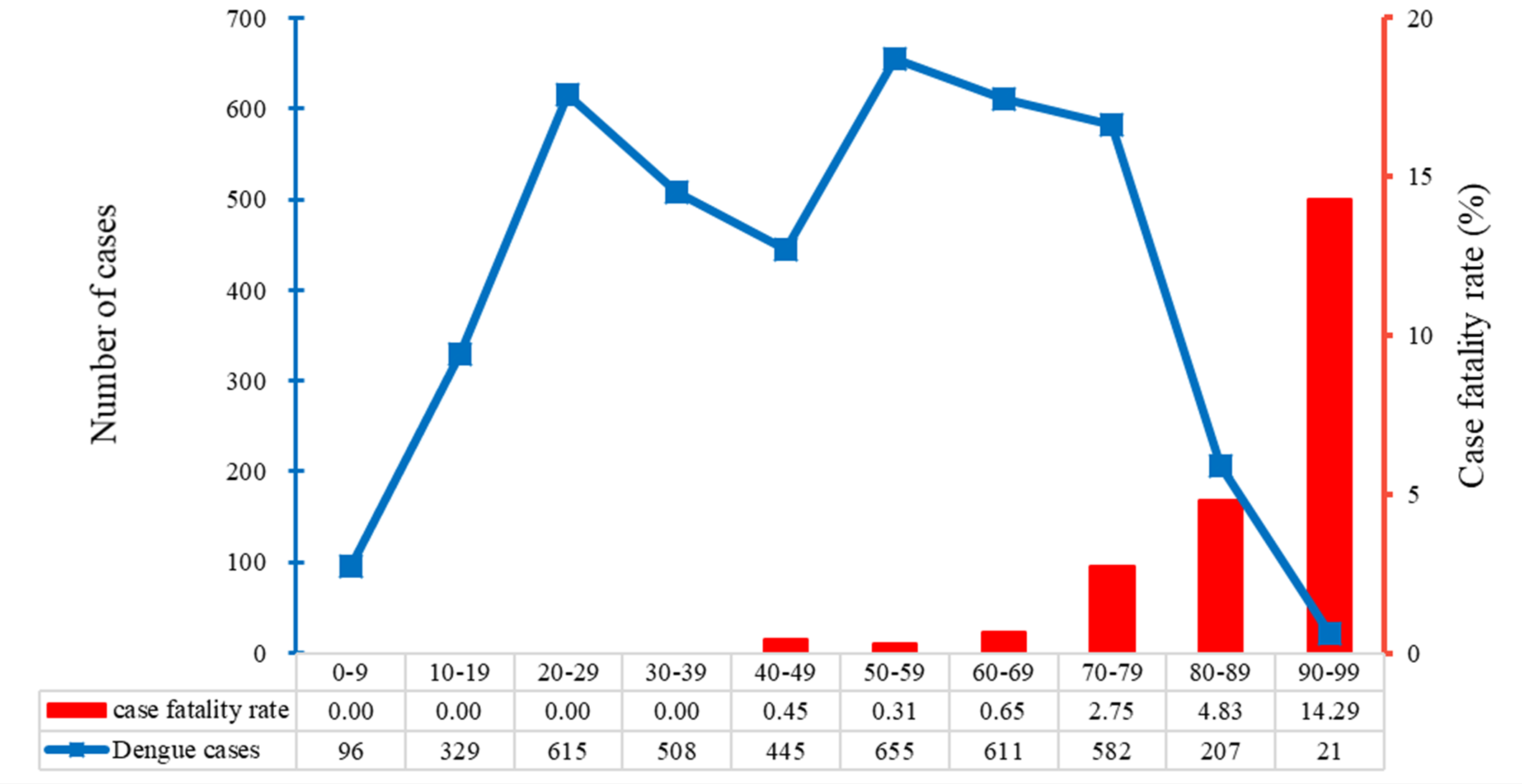

Supplement: S1 Fig — (TIF) [file pntd.0007817.s002.tif]

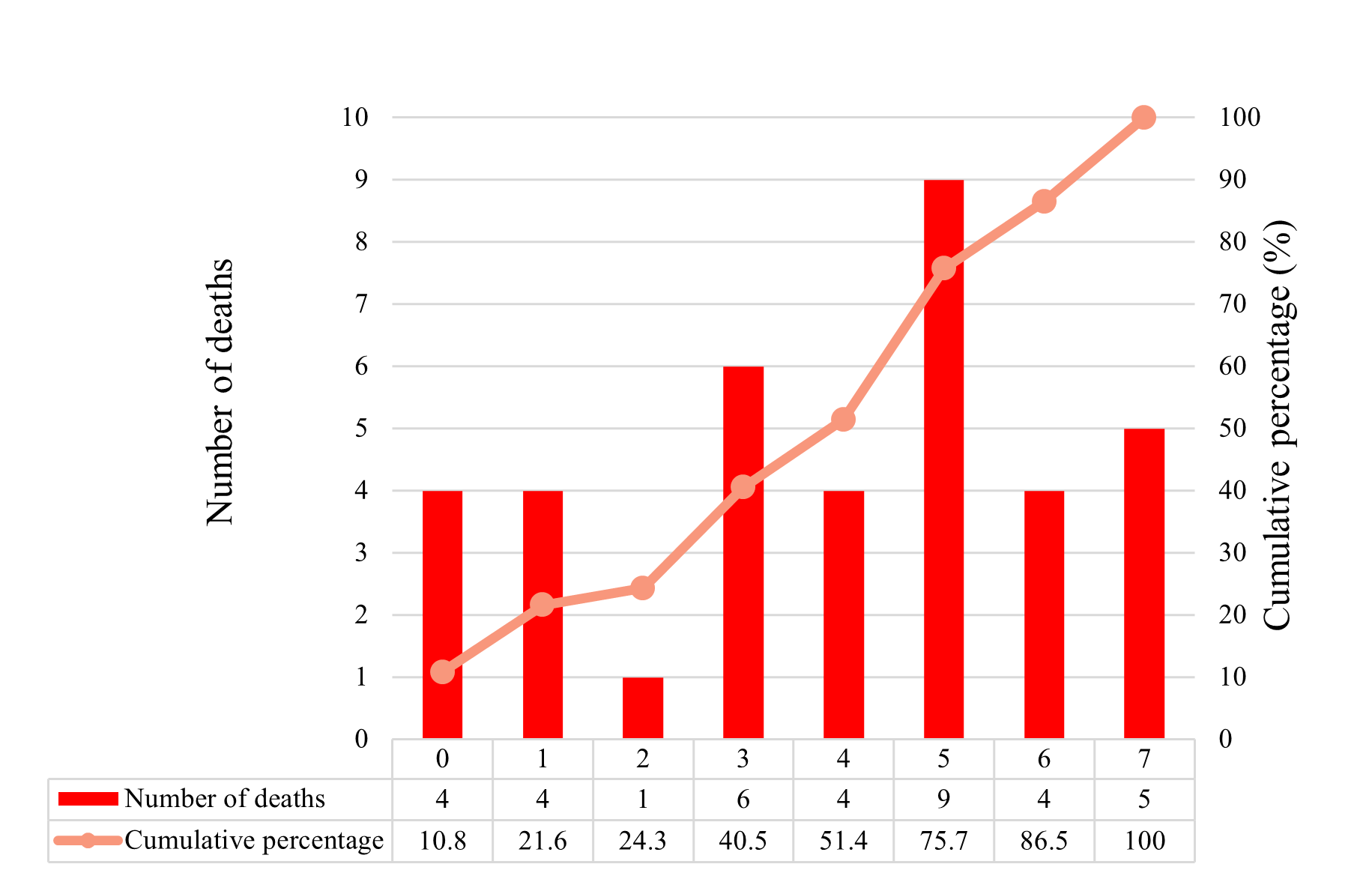

Supplement: S2 Fig — (TIF) [file pntd.0007817.s003.tif]
