# Supplementary material for: Trajectories of hepatic and coagulation dysfunctions related to a rapidly fatal outcome among hospitalized patients with dengue fever in Tainan, 2015
Source: PLoS Negl Trop Dis. 2019 Dec 5;13(12):e0007817. doi: 10.1371/journal.pntd.0007817 (PMC6894745; doi:10.1371/journal.pntd.0007817)
Supplement: S1 Table — (DOCX) [file pntd.0007817.s004.docx]

S1 Table. Factors associated with trajectories of aspartate aminotransferase (AST), alanine transaminase (ALT), activated partial thromboplastin time (aPTT), platelet values of DF patients within 8 days after illness onset.

| **Variables** | | **β** | **Standard Error** | **t value** | ***p*** |
| --- | --- | --- | --- | --- | --- |
| AST | (Intercept) | -124.36 | 31.89 | -3.90 | <0.001 |
|  | Age | 2.92 | 0.52 | 5.62 | <0.001 |
|  | Male | 19.53 | 21.30 | 0.92 | 0.36 |
|  | Fatal | 2812.72 | 110.42 | 25.47 | <0.001 |
|  | Day | 29.52 | 4.00 | 7.39 | <0.001 |
|  | Diagnosis of viral hepatitis B or C | -138.81 | 181.77 | -0.76 | 0.45 |
|  | Diseases of hematological disorders | 56.89 | 48.05 | 1.18 | 0.24 |
|  | Diagnosis of hypertensive disease | -84.03 | 36.94 | -2.28 | 0.02 |
|  | Diagnosis of acute hepatitis or liver failure | 397.07 | 77.74 | 5.11 | <0.001 |
| ALT | (Intercept) | -26.27 | 8.28 | -3.17 | <0.001 |
|  | Age | 0.80 | 0.14 | 5.78 | <0.001 |
|  | Male | 10.68 | 5.55 | 1.92 | 0.05 |
|  | Fatal | 560.39 | 29.23 | 19.17 | <0.001 |
|  | Day | 11.94 | 0.93 | 12.84 | <0.001 |
|  | Diagnosis of viral hepatitis B or C | -48.77 | 47.91 | -1.02 | 0.31 |
|  | Diseases of hematological disorders | 12.04 | 12.54 | 0.96 | 0.34 |
|  | Diagnosis of hypertensive disease | -1.61 | 9.35 | -0.17 | 0.86 |
|  | Diagnosis of acute hepatitis or liver failure | 157.48 | 22.13 | 7.12 | <0.001 |
| aPTT | (Intercept) | 37.26 | 0.58 | 64.13 | <0.001 |
|  | Age | 0.02 | 0.01 | 2.21 | 0.03 |
|  | Male | 1.90 | 0.40 | 4.79 | <0.001 |
|  | Fatal | 12.18 | 1.61 | 7.58 | <0.001 |
|  | Day | 0.40 | 0.09 | 4.46 | <0.001 |
|  | Diagnosis of viral hepatitis B or C | -3.47 | 3.40 | -1.02 | 0.31 |
|  | Diseases of hematological disorders | 2.05 | 0.79 | 2.58 | 0.01 |
|  | Diagnosis of hypertensive disease | -1.49 | 0.70 | -2.12 | 0.03 |
|  | Diagnosis of acute hepatitis or liver failure | 3.16 | 1.35 | 2.34 | 0.02 |
| Platelet | (Intercept) | 220.75 | 3.48 | 63.47 | <0.001 |
|  | Age | -1.09 | 0.06 | -18.84 | <0.001 |
|  | Male | -10.01 | 2.18 | -4.60 | <0.001 |
|  | Fatal | -25.20 | 10.34 | -2.44 | 0.01 |
|  | Day | -13.01 | 0.33 | -39.01 | <0.001 |
|  | Diagnosis of viral hepatitis B or C | 3.45 | 18.19 | 0.19 | 0.85 |
|  | Diseases of hematological disorders | -33.32 | 4.18 | -7.98 | <0.001 |
|  | Diagnosis of hypertensive disease | 3.10 | 3.24 | 0.96 | 0.34 |
|  | Diagnosis of acute hepatitis or liver failure | -24.79 | 7.79 | -3.18 | 0.001 |
