# Supplementary material for: Trajectories of hepatic and coagulation dysfunctions related to a rapidly fatal outcome among hospitalized patients with dengue fever in Tainan, 2015
Source: PLoS Negl Trop Dis. 2019 Dec 5;13(12):e0007817. doi: 10.1371/journal.pntd.0007817 (PMC6894745; doi:10.1371/journal.pntd.0007817)
Supplement: S2 Table — (DOCX) [file pntd.0007817.s005.docx]

S2 Table. The difference of aspartate aminotransferase (AST), alanine transaminase (ALT), activated partial thromboplastin time (aPTT), and platelet values between fatal and survivor group on each day.

| Variable | | Fatal | | | | Survivor | | | | *p* value |
| --- | --- | --- | --- | --- | --- | --- | --- | --- | --- | --- |
|  |  | test results | Mean | SD | Median (range) | test results | Mean | SD | Median (range) |  |
| AST (U/L) | Day 0 | 6 | 443.67 | 554.14 | 185 (47-1481) | 529 | 59.13 | 93.02 | 35 (17-1019) | <0.001 |
|  | Day 1 | 10 | 1059.1 | 2297.88 | 185.5 (65-7438) | 785 | 72.23 | 191.39 | 38 (15-3992) | <0.001 |
|  | Day 2 | 8 | 1440.25 | 2494.5 | 343.5 (48-7407) | 688 | 110.41 | 406.83 | 49 (16-7228) | <0.001 |
|  | Day 3 | 7 | 2327.86 | 3887.83 | 624 (138-10890) | 816 | 141.79 | 431.13 | 60 (18-6022) | <0.001 |
|  | Day 4 | 10 | 3698.6 | 4201.78 | 2011 (176-11741) | 826 | 165.92 | 655.03 | 68 (17-14456) | <0.001 |
|  | Day 5 | 13 | 4696.31 | 4211.9 | 3361 (127-11866) | 753 | 183.8 | 612.17 | 78 (16-10246) | <0.001 |
|  | Day 6 | 5 | 9004.2 | 4840.48 | 9805 (2393-14774) | 739 | 231.11 | 941.51 | 90 (18-14113) | <0.001 |
|  | Day 7 | 1 | 1696 | 0 | 1696 | 668 | 256.09 | 1054.85 | 92 (24-14552) | 0.09 |
| ALT (U/L) | Day 0 | 16 | 70.5 | 107.97 | 33.5 (10-357) | 1105 | 31.27 | 44.35 | 16 (10-491) | 0.10 |
|  | Day 1 | 16 | 364.31 | 811.85 | 96.5 (10-3277) | 1431 | 34.41 | 83.95 | 18 (10-2562) | <0.001 |
|  | Day 2 | 12 | 414.75 | 874.82 | 42.5 (10-3010) | 976 | 50.36 | 150.19 | 23 (10-2459) | 0.15 |
|  | Day 3 | 7 | 710 | 1118.13 | 116 (14-3110) | 1064 | 59.85 | 134.54 | 29 (8-2095) | 0.01 |
|  | Day 4 | 12 | 1197.67 | 1493.91 | 451 (59-4687) | 1012 | 69.55 | 160.8 | 33.5 (10.2461) | <0.001 |
|  | Day 5 | 13 | 1132 | 1197.41 | 659 (50-3401) | 907 | 89.69 | 298.93 | 40 (10-6366) | <0.001 |
|  | Day 6 | 5 | 1845 | 1812.85 | 1504 (377-4940) | 880 | 105.78 | 307.8 | 49 (10-5744) | <0.001 |
|  | Day 7 | 1 | 658 | 0 | 658 | 783 | 110.04 | 276.04 | 52 (10-4641) | 0.1 |
| aPTT (seconds) | Day 0 | 7 | 41.67 | 10.84 | 39.7 (30.9-60.8) | 306 | 38.44 | 6 | 38 (27.5-93.2) | 0.65 |
|  | Day 1 | 7 | 47.4 | 8.39 | 48.1 (35.7-59) | 503 | 38.88 | 5.99 | 38.3 (25.2-91.4) | 0.01 |
|  | Day 2 | 5 | 43.78 | 11.31 | 40.4 (32.7-61.1) | 323 | 41.19 | 6.35 | 40.2 (27.9-80.2) | 0.83 |
|  | Day 3 | 2 | 42.2 | 7.35 | 42.2 (37-47.4) | 312 | 43.08 | 17.14 | 40.8 (30-314.9) | 0.89 |
|  | Day 4 | 3 | 49.3 | 9.32 | 46.5 (41.7-59.7) | 288 | 43.07 | 12.03 | 40.4 (26.2-185.1) | 0.10 |
|  | Day 5 | 12 | 66.01 | 11.16 | 68.7 (52.2-82.1) | 263 | 42.38 | 8.39 | 40.1 (24-78.9) | <0.001 |
|  | Day 6 | 1 | 67.7 | 0 | 67.7 | 241 | 40.42 | 6.85 | 39 (25.8-80.5) | 0.09 |
|  | Day 7 | 1 | 53.1 | 0 | 53.1 | 153 | 39.56 | 8.57 | 38 (29.4-107) | 0.12 |
| Platelet (x10^3^/μl) | Day 0 | 3 | 120 | 104.68 | 113 (19-228) | 546 | 165.4 | 66.24 | 166 (5-567) | 0.38 |
|  | Day 1 | 9 | 143.22 | 101.58 | 125 (13-359) | 688 | 146.69 | 63.21 | 148 (4-369) | 0.59 |
|  | Day 2 | 13 | 74.23 | 68.91 | 79 (7-235) | 410 | 108.88 | 61.07 | 115 (4-345) | 0.03 |
|  | Day 3 | 7 | 28.29 | 21.98 | 19 (9-70) | 389 | 91.1 | 59.25 | 91 (4-325) | <0.01 |
|  | Day 4 | 10 | 33.9 | 36.01 | 19.5 (6-123) | 412 | 75.08 | 58.22 | 63 (5-303) | 0.02 |
|  | Day 5 | 6 | 29 | 28.03 | 14.5 (6-71) | 427 | 61.44 | 49.14 | 48 (4-226) | 0.06 |
|  | Day 6 | 5 | 22.8 | 29.38 | 11 (4-75) | 394 | 62.21 | 49.06 | 50 (5-227) | 0.02 |
|  | Day 7 | 2 | 28 | 31.11 | 28 (6-50) | 361 | 70.96 | 54.15 | 58 (2-325) | 0.18 |

SD=standard deviation
