# Supplementary material for: Trajectories of hepatic and coagulation dysfunctions related to a rapidly fatal outcome among hospitalized patients with dengue fever in Tainan, 2015
Source: PLoS Negl Trop Dis. 2019 Dec 5;13(12):e0007817. doi: 10.1371/journal.pntd.0007817 (PMC6894745; doi:10.1371/journal.pntd.0007817)
Supplement: S3 Table — (DOCX) [file pntd.0007817.s006.docx]

S3 Table. Characteristics of aspartate aminotransferase (AST), alanine transaminase (ALT), AST^2^/ALT, activated partial thromboplastin time (aPTT), and aspartate aminotransferase/platelet count ratio index (APRI) at selected criteria in each day

| Variable | | Criteria | TPR | 1-FPR | TPR-FPR | AUC |
| --- | --- | --- | --- | --- | --- | --- |
| AST (U/L) | Day 0 | 101 | 0.88 | 0.9 | 0.78 | 0.92 |
|  | Day 1 | 98 | 0.93 | 0.87 | 0.80 | 0.94 |
|  | Day 2 | 111 | 0.91 | 0.81 | 0.72 | 0.9 |
|  | Day 3 | 203 | 0.9 | 0.9 | 0.80 | 0.95 |
|  | Day 4 | 225 | 0.92 | 0.89 | 0.82 | 0.96 |
|  | Day 5 | 281 | 0.93 | 0.91 | 0.84 | 0.96 |
|  | Day 6 | 1890 | 1 | 0.98 | 0.98 | 0.99 |
|  | Day 7 | 1627 | 1 | 0.97 | 0.97 | 0.97 |
| ALT(U/L) | Day 0 | 27 | 0.55 | 0.69 | 0.24 | 0.56 |
|  | Day 1 | 60 | 0.68 | 0.87 | 0.56 | 0.81 |
|  | Day 2 | 40 | 0.67 | 0.7 | 0.36 | 0.65 |
|  | Day 3 | 55 | 0.78 | 0.73 | 0.51 | 0.83 |
|  | Day 4 | 81 | 0.81 | 0.81 | 0.62 | 0.91 |
|  | Day 5 | 129 | 0.93 | 0.86 | 0.79 | 0.93 |
|  | Day 6 | 361 | 1 | 0.96 | 0.96 | 0.98 |
|  | Day 7 | 584 | 1 | 0.97 | 0.97 | 0.97 |
| AST^2^/ALT | Day 0 | 360.53 | 0.875 | 0.95 | 0.82 | 0.94 |
|  | Day 1 | 220.16 | 0.92 | 0.87 | 0.79 | 0.95 |
|  | Day 2 | 807.19 | 0.8 | 0.97 | 0.77 | 0.94 |
|  | Day 3 | 337.35 | 1 | 0.84 | 0.84 | 0.95 |
|  | Day 4 | 459.27 | 1 | 0.87 | 0.87 | 0.96 |
|  | Day 5 | 1941.50 | 0.86 | 0.96 | 0.82 | 0.9 |
|  | Day 6 | 7846.61 | 1 | 0.98 | 0.98 | 1 |
|  | Day 7 | 4255.16 | 1 | 0.98 | 0.98 | 0.98 |
| aPTT (seconds) | Day 0 | 39.6 | 0.57 | 0.63 | 0.20 | 0.54 |
|  | Day 1 | 43.6 | 0.78 | 0.84 | 0.62 | 0.84 |
|  | Day 2 | 48.3 | 0.4 | 0.89 | 0.29 | 0.52 |
|  | Day 3 | 47.3 | 0.5 | 0.81 | 0.31 | 0.51 |
|  | Day 4 | 46.2 | 0.75 | 0.76 | 0.51 | 0.76 |
|  | Day 5 | 52 | 1 | 0.88 | 0.88 | 0.95 |
|  | Day 6 | 64.1 | 1 | 0.99 | 0.99 | 0.99 |
|  | Day 7 | 52.7 | 1 | 0.94 | 0.94 | 0.94 |
| APRI | Day 0 | 5.47 | 1 | 0.97 | 0.97 | 0.98 |
|  | Day 1 | 1.16 | 0.8 | 0.81 | 0.61 | 0.84 |
|  | Day 2 | 6.3 | 0.83 | 0.86 | 0.69 | 0.91 |
|  | Day 3 | 19.18 | 1 | 0.84 | 0.84 | 0.96 |
|  | Day 4 | 93.4 | 0.67 | 0.96 | 0.63 | 0.86 |
|  | Day 5 | 27.31 | 1 | 0.87 | 0.87 | 0.96 |
|  | Day 6 | 157.11 | 1 | 0.95 | 0.95 | 0.98 |
|  | Day 7 | 200.17 | 1 | 0.97 | 0.97 | 0.97 |

Abbreviations: AUC, area under the curve; CI, confidence interval; AST, aspartate aminotransferase; ALT, alanine transaminase; aPTT, activated partial thromboplastin time; APRI, aspartate aminotransferase/platelet count ratio index; TPR, true positive rate; FPR, false positive rate.
